# Different trunk muscle responses to unexpected balance perturbations between older recurrent fallers and older non-fallers: a combined wearable ultrasound imaging and electromyography (EMG) study

**DOI:** 10.1186/s12877-026-07158-7

**Published:** 2026-02-12

**Authors:** Hao-Bin Liang, Ringo Tang-Long Zhu, Yu-Yan Luo, Zhen Song, Wei-Tao He, Isabella Chee-Yin Yam, Ka-Shing Lee, Wei Ren, Ke-Jing Li, Feng-Yi Wang, Shuai Li, Huiying Cynthia Hou, Quan Wei, Yong-Ping Zheng, Xiu-Qin Ye, Christina Zong-Hao Ma

**Affiliations:** 1 Department of Biomedical Engineering, The Hong Kong Polytechnic University, Hung Hom, 999077 Hong Kong SAR, China; 2 Research Institute for Smart Ageing, The Hong Kong Polytechnic University, Hung Hom, 999077 Hong Kong SAR, China; 3 Department of Rehabilitation, Southwest Hospital, Third Military Medical University (Army Medical University), Chongqing, 400038 China; 4 Department of Rehabilitation Sciences, The Hong Kong Polytechnic University, Hung Hom, 999077 Hong Kong SAR, China; 5 Department of Computer Science, City University of Hong Kong, Hung Hom, 999077 Hong Kong SAR, China; 6 Rehabilitation Medicine Center, Institute of Rehabilitation Medicine, West China Hospital, Sichuan University, Chengdu, 610041 China; 7 Key Laboratory of Rehabilitation Medicine in Sichuan Province, West China Hospital, Sichuan University, Chengdu, 610041 China; 8 Department of Building Environment and Energy Engineering, The Hong Kong Polytechnic University, Hung Hom, Hong Kong SAR, China; 9 Ultrasound Department, Shenzhen People’s Hospital, The Second Clinical Medical College of Jinan University, Shenzhen, 518020 China

**Keywords:** Falls, Older people, Reactive balance, Sonomyography (SMG), Abdominal muscle, Muscle thickness, Morphological, Contractile, Neuromuscular, Kinematic

## Abstract

**Background:**

Falls are important public health issues among older people. Previous studies reported the fall-related alterations in electromyographic (EMG) activation of specific trunk muscles following a single direction of perturbation, while the activation patterns following multiple directions of balance perturbations remained unclear. Additionally, although the static structural/morphological characteristics of trunk muscles have been associated with balance performance, the fall-related trunk muscle contraction patterns for maintaining reactive balance remained unknown. This pilot observational study therefore aimed to comprehensively explore how trunk muscles’ activation and contraction patterns during reactive balance control differed between older recurrent fallers and older non-fallers.

**Methods:**

Six community-dwelling older recurrent fallers (70.0 ± 5.1 years; 4 females; ≥2 falls in past one year), and six older non-fallers (70.8 ± 3.9 years; 5 females) were recruited. Eligibility criteria were aged 65 years or older, with independent mobility, without cognitive impairment or balance-affecting conditions, without recent injuries/surgery, and without recent structured exercise. Participants received unpredictable translational moving-platform balance perturbations during natural standing. The dominant-side trunk muscle thickness changes (measured by wearable ultrasound imaging) and electrical activities (measured by EMG) for maintaining reactive balance were focused, supplemented by analyzing pelvic motions and postural sways.

**Results:**

Compared to older non-fallers, older recurrent fallers had significantly: (1) 51% smaller rate (following anterior perturbations; d = 1.47; *p* = 0.029) and 50% smaller peak (following medial perturbations; d = 1.32; *p* = 0.045) of the internal oblique (IO) muscle thickness change; and (2) smaller peak of IO muscle EMG signal following medial perturbations, and slower/smaller EMG responses of the rectus abdominis (RA) muscle following posterior/medial/lateral perturbations (*p* < 0.05).

**Conclusions:**

This study demonstrated the feasibility of using wearable dynamic ultrasound imaging to characterize fall-related morphological changes during reactive balance control in older adults. The participated recurrent fallers exhibited the perturbation-direction-specific slower/smaller activation and contraction of IO muscle and slower/smaller activation of RA muscle, as compared to non-fallers. Given the pilot study, future longitudinal research with adequately powered samples is warranted to confirm this preliminary finding, which may help complement the current fall-risk assessments and imply the targeted fall-prevention exercises in older adults.

**Supplementary Information:**

The online version contains supplementary material available at https://doi.org/10.1186/s12877-026-07158-7.

## Introduction

Falls are important public health issues among older people [1]. It was estimated that 684,000 people died from falls each year, and 37.3 million severe falls required medical attention each year [2]. Muscle weakness and balance disorder have been major risk factors for falls in the older adults [3]. In addition to lower limb muscles [4–6], the strength of trunk muscles is also crucial for maintaining volitional balance [7–10]. Moreover, some falls in older adults are caused by external perturbations (such as tripping or slipping), requiring prompt and adequate reactive balance control [11, 12]. Although the importance of the trunk muscles in maintaining balance has been validated [7], their responses and specific function during reactive balance control remain relatively underexplored.

Several prior studies have investigated the age-related [13, 14] or fall-related [14, 15] alterations of trunk muscle activation during reactive balance control. Two studies reported that older adults exhibited delayed electromyographic (EMG) onset of trunk muscles [i.e., rectus abdominis (RA), erector spinae (ES), external oblique (EO)], as compared to young adults in maintaining reactive balance [13, 14]. However, these identified age-related different trunk muscle activation for maintaining reactive balance may not be able to directly reflect the reasons for increased fall risk in older people. It would be necessary to exclude the factor/influence of age and specifically compare the differences in trunk muscle function among the older adults, between those with and without a history of falls (i.e., older fallers vs. older non-fallers), to directly investigate the impact of falls. Two previous studies reported that older fallers exhibited delayed EMG onset and distinct activation sequences of trunk muscles, as compared to older non-fallers, in response to the unexpected left balance losses induced by right shoulder-impact perturbations [14, 15]. However, they only focused on the responses following single direction of perturbations, and analyzed only a few EMG parameters (i.e., EMG integral, EMG onset latency) [14, 15]. Trunk muscles’ responses to the more complex anterior, posterior, and/or left perturbations have remained unclear. A more comprehensive analysis of trunk muscle activities following more complexed perturbations that were related to falls is needed, including utilizing some additional EMG parameters and simulating multidirectional perturbations. This will help reveal the fall-prone older people’s specific decline or even impairment in trunk muscles that leads to their poorer reactive balance control.

In addition to the trunk muscle strength as measured by a dynamometer [7–10] and the muscle activation as measured by EMG sensors [13, 14, 16], previous studies have also indicated that the internal structure or morphology (i.e., muscle density, thickness, cross-sectional area) of trunk muscles was associated with balance performance (i.e., postural sway during static standing and functional mobility) in older adults [17–19]. However, these studies have primarily focused on the relationship between static trunk muscle morphology and static balance. There is still a lack of sufficient evidence regarding the role of dynamic changes in trunk muscle morphology during reactive balance tasks. Following unexpected perturbations, although the prompt muscle activation is necessary to generate fall-avoidance strategies [20], the onset of muscle’s electrical activation does not instantaneously translate into mechanical force generation [21]. The time lag between electrical activation and mechanical contraction (i.e., activation-contraction delay) may affect the reactive speed of balance-control strategy [21]. It is therefore necessary to adopt some more advanced technologies to investigate and compare the difference in trunk muscles’ real-time contraction patterns or structural changes, during dynamic reactive balance maintaining situations, between older fallers and older non-fallers. However, possibly due to some technical limitations, the muscle activation-contraction delays have only been investigated in the sitting conditions (e.g., voluntary contraction of a leg muscle) [21–25].

With the advancement of wearable technology, some novel wearable ultrasound imaging devices have been emerging to record the real-time muscle contraction patterns and morphological changes in more dynamic walking situations [26–35]. It would be interesting to utilize the wearable B-mode ultrasound imaging technology to study and compare the real-time internal morphological changes, contraction sequence, and activation-contraction delays of trunk muscles between older fallers and older non-fallers during perturbations. This may also help identify the fall-related alterations of trunk muscles underlying poorer reactive balance control, and facilitate the development of more sensitive and reliable fall-risk assessments.

Therefore, this pilot study aimed to investigate and compare the differences in trunk muscle thickness changes and EMG signals, following unexpected moving-platform perturbations, between older recurrent fallers and older non-fallers. The three-dimensional pelvic motions and postural sways between the two groups were also compared, to complement and validate the analysis of muscular responses. Both the temporal and amplitude parameters were analyzed for all signals to achieve a comprehensive investigation [20].

Based on the older fallers’ deficits in reactive balance control as reported in prior studies, it was hypothesized that following balance perturbations from any of the four directions (i.e., anterior/posterior/medial/lateral), older recurrent fallers would show the below alterations as compared to older non-fallers in trunk muscles:

- Slower and smaller thickness changes and EMG responses, along with larger activation-contraction delays (confirmatory hypothesis);
- Different activation or contraction patterns (exploratory hypothesis).

It is expected that the outcomes of this study can provide a more comprehensive understanding of reactive balance control strategies in fall-prone older adults, by focusing on the trunk muscles’ activation and contraction. This may help facilitate the development of some more specialized trunk muscle screening and training programs, to reduce fall risk and fall incidence of older people in future clinical practice.

## Methods

### Study design

A pilot cross-sectional study was conducted on two participant groups of (1) older recurrent fallers and (2) older non-fallers.

### Participants

A total of 12 community-dwelling older participants (6 recurrent fallers and 6 non-fallers) were recruited through convenience sampling, via advertisements in local community centers. The recruitment period was from July 2024 to November 2024. Before being tested, each participant read and signed an informed consent form to participate in this study (Ethics approval agency: Institutional Review Board, The Hong Kong Polytechnic University; Ethical reference number: HSEARS20240604004; Date of approval: 4 June 2024).

A fall was defined as an unintended event that led to a person coming to the ground or a lower level, and was not caused by intrinsic factors or significant hazards [36]. The inclusion criteria were: (1) aged ≥ 65 years old [37]; (2) recurrent fallers (with ≥ 2 falls within the past one year) [38] and non-fallers (with no fall within the past one year); (3) able to independently come to the laboratory without any assistance; and (4) with no cognitive impairment as measured by the Montreal Cognitive Assessment Hong Kong Version (HK-MoCA), i.e., age- and education-adjusted score>16th percentile [39]. Exclusion criteria were: (1) having conditions such as diabetes, vestibular disorders, heart diseases, severe osteoporosis, or a history of stroke that may affect balance and limit physical activities [12]; (2) currently experiencing unresolved injuries from a fall, having undergone surgery within the past year without full recovery, or currently residing in a hospital requiring monitored treatment; and (3) having participated in any structured exercise or strengthening regimen (rather than general daily physical activity) within the past half year [40, 41]. All eligible participants were informed about the procedures in advance, which consisted of subjective assessments and perturbation trials, before signing the informed consent form.

### Subjective assessments

The demographic data were collected from each participant first, including age, sex, body mass, height, medical history, and fall history. The following questionnaires and scales were then used to complete the remaining subjective assessments. The Falls Efficacy Scale-International (FES-I) short version was used to investigate participant’s fear of falling [42]. The HK-MoCA was adopted to evaluate each participant’s cognitive level [43]. The Chinese Version of the Physical Activity Scale for the Elderly (PASE-C) was used to survey each participant’s level of activity over the past seven days [44]. The Mini-Balance Evaluation System Test (Mini-BEST) was performed to assess each participant’s balance capability [45]. The Mini-BEST was selected based on its established reliability and validity, and its comprehensive evaluation of various balance dimensions, particularly for the sub-item of reactive balance, making it more relevant to the focus of this study than other clinical assessments (e.g., Timed Up and Go test, Berg Balance Scale) [20, 46]. The rationale for using these clinical questionnaires, scales, or tests was because fear of falling, cognitive level, physical activity level, and overall balance performance are known relevant to fall risks [47]. It is necessary to measure and account for these possible confounding variables. Finally, participants were asked, “If you shoot a ball on a target, which leg would you use to shoot the ball?”, to determine their dominant side [48]. The data collection was conducted offline in the laboratory by a single trained evaluator to eliminate inter-rater variability, and participation was completely voluntary and anonymous.

### Perturbation trials

**Fig. 1:**
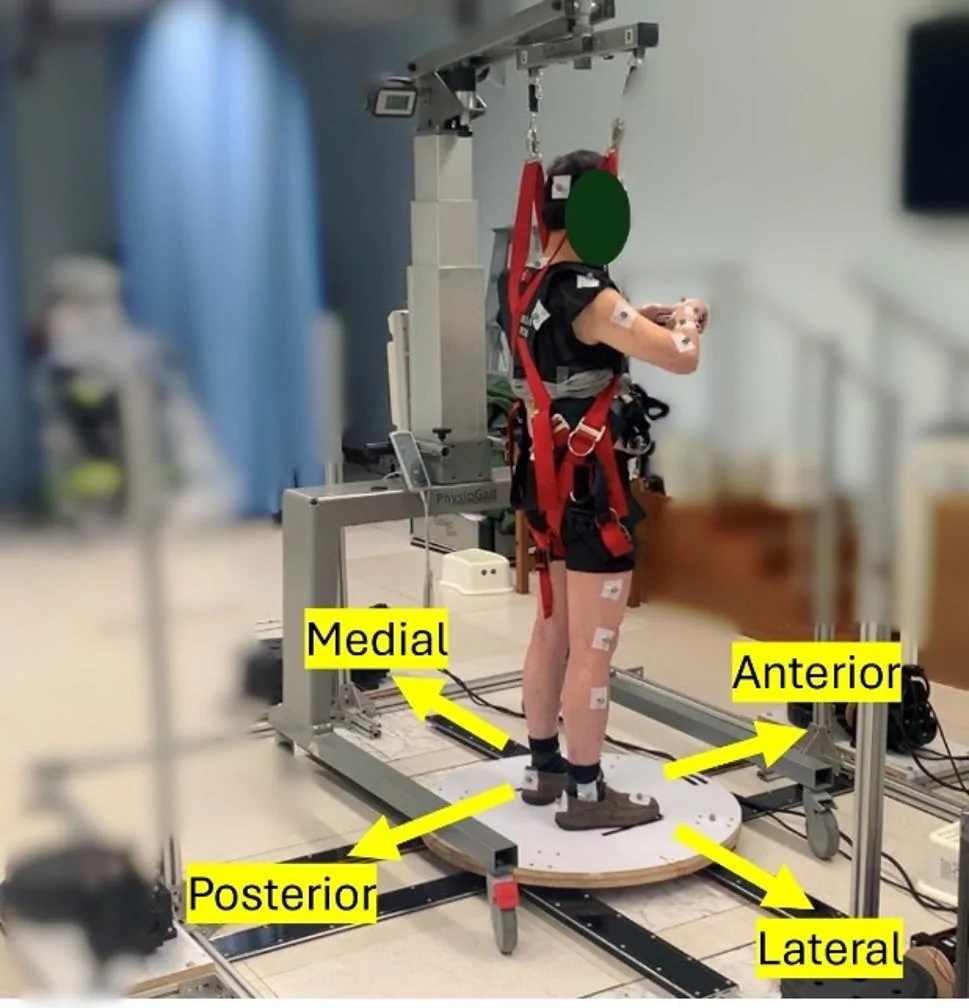
Illustration of the moving platform system with a participant

A previously validated reliable moving-platform system was used to provide participants with unexpected translational perturbations and induce their reactive balance control (Fig. 1) [49]. A perturbation was defined as the sudden displacement of the moving platform that occurred randomly towards the anterior, posterior, medial, or lateral direction relative to the participant’s dominant leg, and would lead to participant’s backward, forward, lateral, or medial balance loss, respectively. Based on previous works, the anterior, posterior, medial, and lateral displacements of perturbations were set as 2.67%, 4%, 5.33%, and 5.33% of the participant’s height, respectively [49]. An eight-channel Trigno Wireless Biofeedback System (Delsys Inc, Natick, MA, USA) that sampled at 2000 Hz was used to collect muscles’ electrical signals. A two-channel wearable wireless ultrasound imaging system was used to capture real-time muscle images (frequency: 20 Hz; imaging width: 38.4 mm, depth: 60 mm; bandwidth: 7.5 MHz ± 35%; dynamic range: 40 dB; pre-set averaged gain + 40 dB) [27]. A three-dimensional (3D) eight-camera motion capture system (Nexus 2.11, Vicon Motion Systems Ltd., Yarnton, UK) was employed to collect the whole-body kinematic data. All measurement systems were synchronized with the perturbation system.

**Fig. 2:**
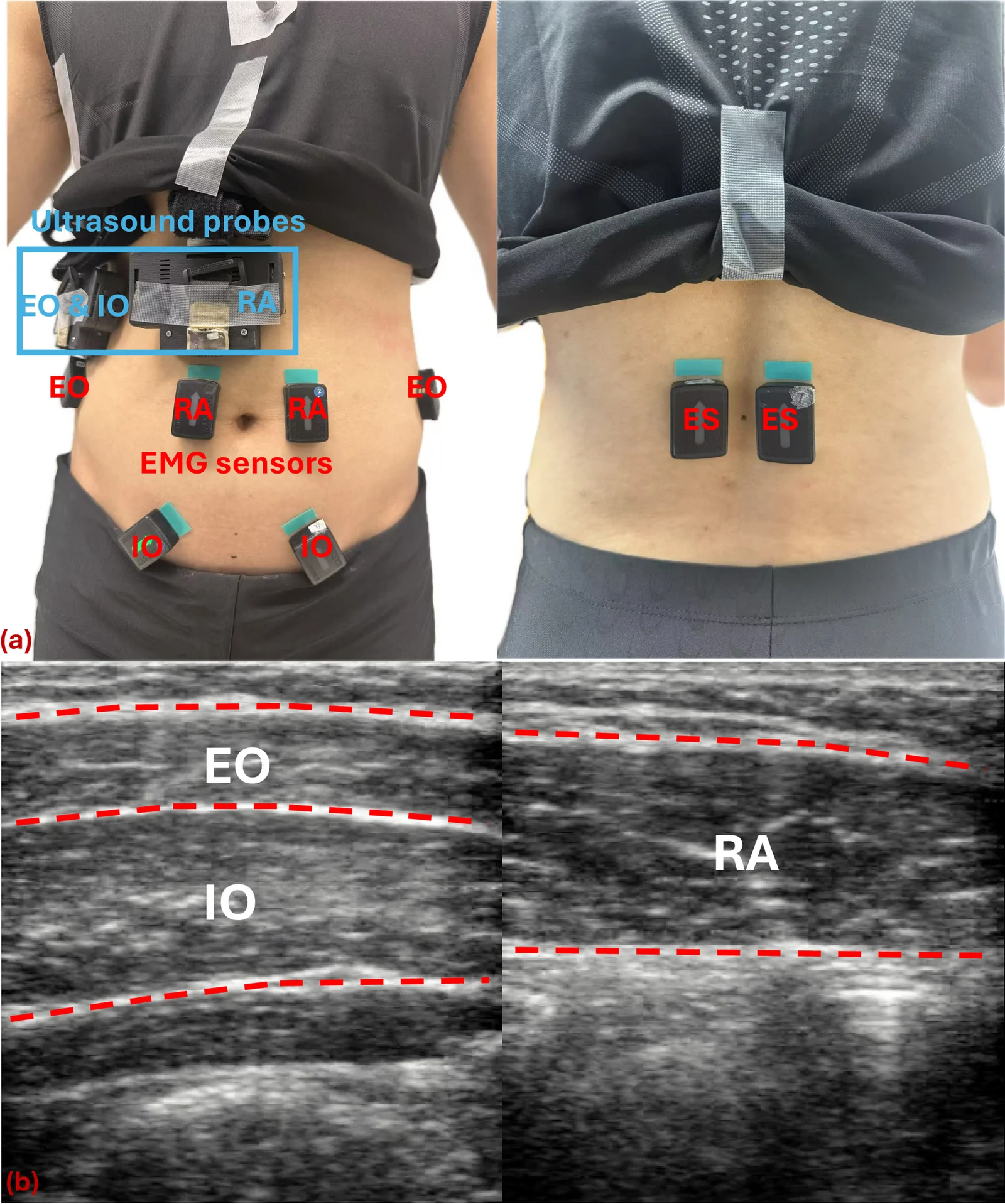
Placement of sensors and sample ultrasound images. **a** Illustration of the placement of two wearable wireless ultrasound probes and eight EMG sensors on a participant’s trunk. **b** An example of the captured ultrasound images of the dominant-side RA, EO and IO muscles. Note: RA: Rectus Abdominis; EO: External Oblique; IO: Internal Oblique, ES: Erector Spinae

Prior to the perturbation trials, participants were instructed to wear comfortable sports or casual shoes, and to avoid sandals, high heels, or boots for safety reasons. Each participant was provided with standardized tight-fitting shirts and shorts to facilitate sensor placement. The EMG sensors, retroreflective markers, and ultrasound probes were then affixed to each participant (Fig. 2). Eight wireless surface EMG sensors were placed on participant’s trunk muscles associated with maintaining postural balance, i.e., bilateral RA muscles, EO muscles, internal oblique (IO) muscles, and ES muscles. The EMG sensors for measuring RA muscles were placed 3-cm lateral to the umbilicus [50], and those for the EO muscles were placed 15-cm lateral to the midline and along the direction of the muscle fibers [51]. The EMG sensors for the IO muscles were positioned 2-cm below the iliac crest in a horizontal position; within the triangle that formed by the inguinal ligament, the external edge of the rectus abdominis sheath, and the line connecting the two anterior superior iliac spines [52]. The EMG sensors for the ES muscles were placed 2-cm lateral to the spinous processes of the fifth lumber vertebra (L5) level [51]. Two wearable wireless ultrasound probes were then placed next to the corresponding EMG sensor on the dominant-side RA (to capture images of RA muscle) and EO (to capture images of the superficial-layer EO muscle and the deep-layer IO muscle) muscles, separately. Specifically, the ultrasound probe for RA muscle was placed lateral to the umbilicus; and that for the EO muscle and IO muscle was placed in the mid-axillary line, between the subcostal line and the iliac crest [31]. Only these three dominant-side abdominal muscles’ ultrasound images were collected, due to the inability to fully capture images of the ES muscle with this wireless wearable equipment and the limited number of synchronized ultrasound probes. Finally, a set of 39 retroreflective markers were attached to each participant’s head, trunk, bilateral upper limbs, pelvis, and bilateral lower limbs at bony landmarks, according to the Plug-in-Gait full-body model. To capture the participants’ natural reactive balance responses and minimize the potential learning or anticipatory effects, no familiarization or practice perturbations were provided to participants prior to the formal data collection.

Each participant was then instructed to stand naturally with two feet shoulder-width apart in the center of the platform, with the original foot position marked with black lines. The participant was asked to hold a short stick to prevent their hands from obstructing the capture of the retroreflective markers on their bodies. The participant was informed to: (1) avoid moving during natural standing; (2) try to maintain balance, upon feeling the perturbation; and (3) try to quickly return to the marked original foot position, if making a step. For safety reasons, each participant was equipped with a safety harness system (PG-360, Physio Gait Dynamic Unweighting System, Healthcare International Ltd., Langley, WA, USA) during all perturbation trials.

Each participant experienced a total of 20 perturbations in two rounds/trials, with each trial consisting of ten perturbations. Each perturbation direction (i.e., anterior, posterior, medial, or lateral) was assigned with five repetitions to form the 20 perturbations. There was a five-minute rest period between the two trials. The timing and direction of the perturbations were randomized and blinded to each participant. Additionally, the camera of a mobile phone was used to video record the entire perturbation trials for later verification of data.

### Data processing

Retroreflective marker trajectories were first processed using the Plug-in-Gait full-body model to obtain kinematic data. Aponeuroses (i.e., muscle boundaries) were annotated on the collected ultrasound images automatically and manually, by using a validated self-developed SMG-master software (version 1.0) to obtain raw thickness values [27, 30, 53]. The kinematic data, raw EMG data, and raw ultrasound muscle thickness data were then further processed as below in the MATLAB program (MATLAB, The MathWorks, Inc., Natick, MA, USA). For each of the processed signals, the onset, peak, offset points were identified as: (1) *Onset*: the first time point following a perturbation when the signal value exceeded the baseline + 2*SD for EMG signals and ultrasound data, as well as the first time point following perturbations when the kinematic data exceeded or dropped below the baseline ± 5*SD [54, 55]; (2) *Peak*: the point with maximum or minimum signal value following the onset (with the onset and peak points been identified within the two seconds after a perturbation, given that participants’ center of mass (COM) displacements could recover to the baseline values within this period [54, 55]); (3) *Offset*: the first time point when the signal value dropped below or exceeded the corresponding value of the onset time point [49, 56] (with the baseline referred to as the mean signal value for the 1000 ms before each perturbation). Based on these identified onsets, peak, and offset points, the temporal (onset latency, time to peak, and burst duration) and amplitude (peak amplitude, rate of rise, or co-contraction index [CCI]) parameters were analyzed for each signal. For each parameter, the average values from the five repetitive perturbations with the same direction were used for statistical analysis. Given the low prevalence of missing onsets (< 10% of perturbations), a physiologically grounded imputation strategy derived from the COM data (restored balance within 2000 ms following perturbations) was applied, the specifics of which are described below [20].

#### Kinematic data processing

The COM displacements and dominant-side pelvic motions were analyzed. To obtain the COM displacement that was relative to the participant’s base of support, the displacement of the moving platform was subtracted from the COM displacement. These kinematic data were further subtracted by the baseline signal value before each perturbation.

The following four temporal and amplitude parameters were then analyzed: (1) *Onset latency*: interval from the start of perturbation to the onset point; (2) *Time to peak*: interval from the start of perturbation to the peak point; (3) *Burst duration*: interval between the onset and offset points (for signals without an onset, the onset latency, time to peak, and burst duration were determined as 2000 ms, 2000 ms, and 0 ms, respectively); and (4) *Peak amplitude*: signal value of the peak point (for signal without onset, the peak amplitude was determined as 0) [49, 56].

#### EMG data processing

The raw EMG data were zeroed to the mean value of the entire perturbation trial first, then full-wave rectified, and low-pass filtered using a bi-directional fourth-order Butterworth filter at the cut-off frequency of 4 Hz. Finally, the signals were normalized by dividing the values by the mean signal values of the 1000-ms duration that was captured before each trial [49, 56].

**Fig. 3:**
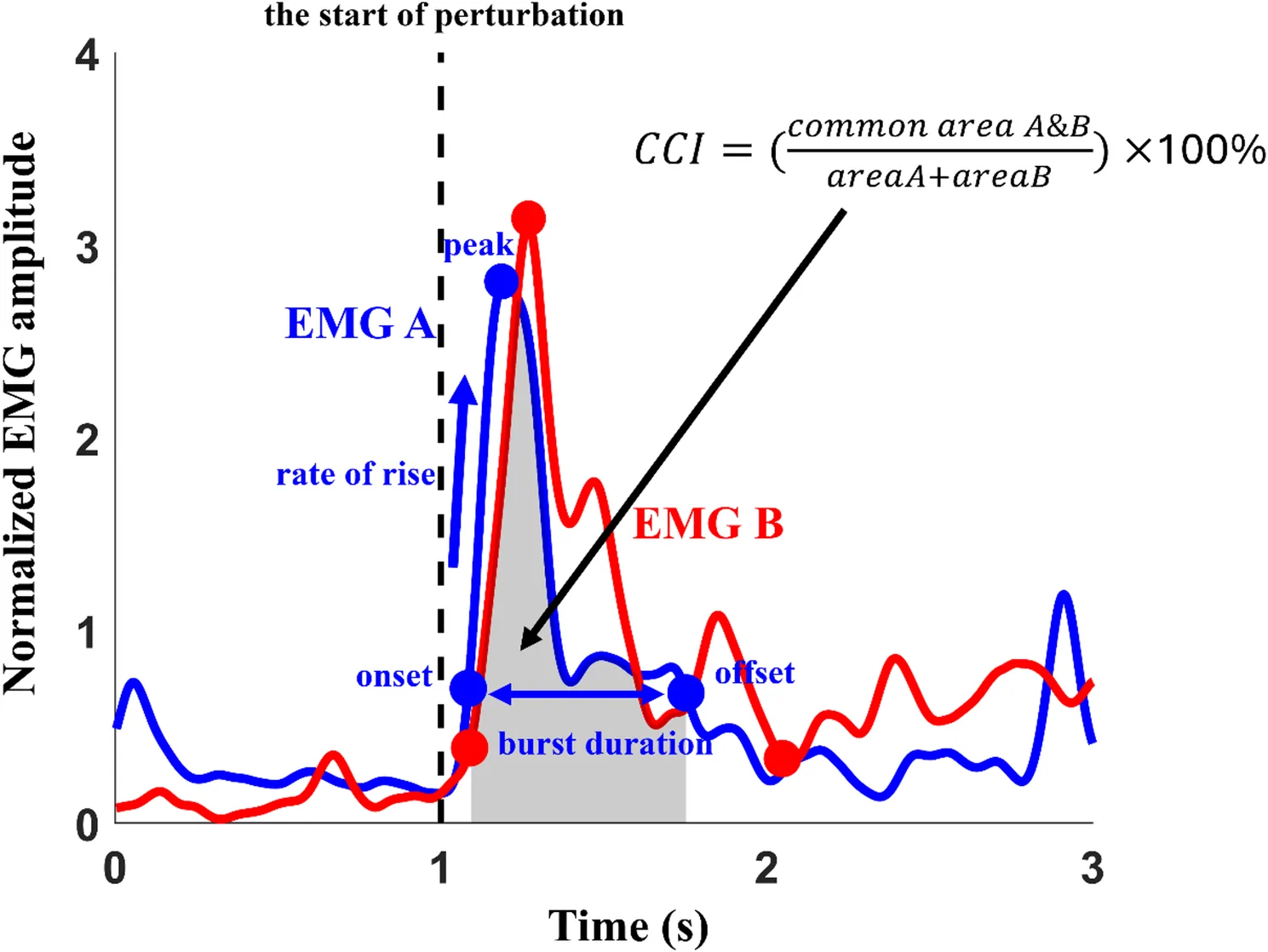
Illustration of the analyzed temporal and amplitude parameters Note: CCI: Co-contraction Index; EMG: Electromyographic

Similar as above, the following six temporal and amplitude parameters were analyzed: (1) *Onset latency* (same as process of kinematic signals); (2) *Time to peak* (same as process of kinematic signals); (3) *Burst duration* (same as process of kinematic signals); (4) *Peak amplitude*: signal value of the peak point (for signal without onset, the peak amplitude was determined as the baseline signal value before the perturbation); (5) *Rate of rise*: gradient of the signal that changed from onset to peak (for signal without onset, the rate of rise was set to 0); and (6) *CCI*: percentage of the overlapping EMG signal area to the total EMG signal area for an agonist-antagonist muscle pair. The CCI was calculated within the duration from two muscles’ later EMG onset to two muscles’ earlier EMG offset, based on the formula in Fig. 3 [49, 56–59].

#### Ultrasound imaging data processing

**Fig. 4:**
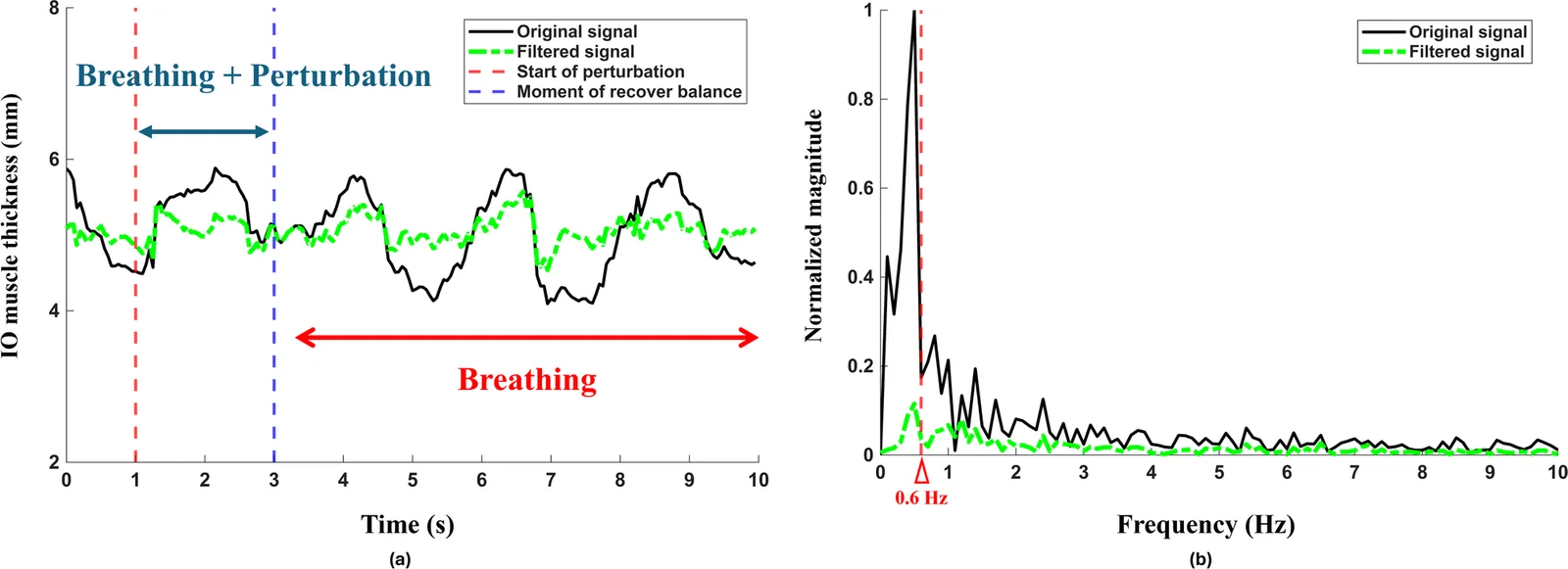
The results of filtering out the breathing noise from the signal of muscle thickness change. **a** A typical signal of muscle thickness change, with and without the filtering; and (**b**) The Fast Fourier Transform spectra of the original signal of muscle thickness change, and the signal after filtered with 0.6 Hz high-pass filter. Note: IO: Internal Oblique

Out of our anticipation, the changes in abdominal muscle thickness were found to be influenced by participant’s breathing activity during the experiment. It was therefore essential to differentiate the abdominal muscle contraction due to perturbation from that due to natural breathing activity. The COM displacement data indicated that participants could recover balance within 2 s following the perturbations. Within the subsequent 3 to 10 s, the participants remained in a static standing position, during which the abdominal muscle contraction was considered primarily due to breathing. After checking the thickness changes within this period (i.e., the subsequent 3 to 10 s), the observed muscle contraction cycle was approximately 2 s, namely that the muscle contraction frequency due to breathing was approximately 0.5 Hz. By further performing a Fast Fourier Transform (FFT) on the original ultrasound imaging signal, the main signal intensity caused by breathing was concentrated in the frequency less than 0.6 Hz. Therefore, a zero-phase second-order high-pass Butterworth filter at 0.6 Hz was used to filter out the noise caused by breathing (Fig. 4). Then the filtered muscle thickness data were subtracted by the baseline value before each perturbation, and further divided by baseline value to reflect the percentage of thickness change relative to baseline.

Similar as above, the following five temporal and amplitude parameters were analyzed: (1) *Onset latency* (same as process of kinematic signals); (2) *Time to peak* (same as process of kinematic signals); (3) *Burst duration* (same as process of kinematic signals); (4) *Peak amplitude* (same as process of kinematic signals); and (5) *Rate of rise* (same as process of EMG signal).

### Statistical analyses

Statistical analyses were conducted using IBM SPSS Statistics 25.0 software package (IBM Corp; Armonk, NY, USA), with a significance level of 0.05.

For the between-group comparisons between older recurrent fallers and older non-fallers, all parameters including demographic data, COM displacements, dominant-side pelvic motions, trunk muscle EMG signals, muscle thickness changes and activation-contraction delays (i.e., onset latency of muscle thickness increase subtracted by that of EMG signal) were analyzed using either the independent sample t-tests (for normally distributed data) or the Mann-Whitney U tests (for non-normally distributed data). Noted that the *p* values for these between-group comparisons (T-test or Mann-Whitney U test) were not corrected. This was due to the specific confirmatory hypothesis tested, namely that the slower and smaller thickness changes and EMG responses along with the larger activation-contraction delays in recurrent fallers were expected. Regarding the between-group differences in reactive balance responses, percentage difference (defined as the mean value of recurrent faller group minus that of non-faller group, and divided by that of non-faller group), mean difference, and effect size (d for independent sample t-test, r for Mann-Whitney U test) were also calculated.

Within the recurrent faller or non-faller group, comparisons among the muscles, between the joint motions around an axis, or between the postural sway directions along an axis were conducted using the methods below. The dominant-side’s trunk muscle thickness change and EMG signal parameters (i.e., onset latency, time to peak, burst duration, peak amplitude, rate of rise, and CCI) were examined using the Friedman tests to investigate differences among muscles, followed by post hoc pairwise comparisons with Bonferroni corrections, as most of these parameters did not normally distribute. The activation-contraction delays were analyzed using one-way repeated ANOVA to investigate differences among muscles, followed by post hoc pairwise comparisons with Bonferroni corrections, as the data of this parameter were following normal distributions. The paired t-tests (for normally distributed data) or the Wilcoxon tests (for non-normally distributed data) were used to investigate difference in the COM displacement on the same axis (i.e., along sagittal axis: forward vs. backward; along frontal axis: medial vs. lateral; and along vertical axis: upward vs. downward) and dominant-side’s pelvic motion parameters on the same axis (i.e., pelvic tilt around frontal axis: anterior vs. posterior tilt; pelvic obliquity around sagittal axis: hike vs. drop; and pelvic rotation around vertical axis: forward vs. backward rotation). Noted that post-hoc pairwise comparisons for one-way repeated measures ANOVA and Friedman tests were Bonferroni-corrected; in contrast, the *p* values of paired t-tests and Wilcoxon tests were not corrected, as they involved comparisons between only two levels.

## Results

### Subjective assessments

As shown in Table 1, there were no significant differences in medication usage, age, body mass, height, BMI, Mini-BEST score, PASE-C score, or HK-MOCA score between older recurrent fallers and older non-fallers. The Short FES-I score of recurrent fallers was significantly higher than that of non-fallers (*p* < 0.05). No adverse events were reported throughout the experiments.

**Table 1:** Subjective assessment results of 6 older recurrent fallers and 6 older non-fallers

Frequency of falls	Recurrent Fallers (4 females & 2 males)	Non-fallers (5 females & 1 male)	Significance
	2 ± 0	0	/
Number of medications	1.0 (1.0)	1.0 (0)	0.485
Age (year)	70.0 ± 5.1	70.8 ± 3.9	0.757
Body mass (kg)	58.1 ± 12.1	50.1 ± 4.0	0.272
Height (cm)	159.6 ± 8.3	157.5 ± 5.1	0.615
BMI (kg/m²)	22.7 ± 4.0	20.3 ± 2.0	0.397
Dominant leg (right/left)	6/0	6/0	/
Short FES-I (score)	15.0 (3.0)	7.0 (1.0)	0.003*
PASE-C (score)	159.4 ± 45.0	149.5 ± 41.0	0.700
Mini-BEST (score)	23.3 ± 2.5	24 ± 1.4	0.852
HK-MOCA (score)	28.7 ± 1.2	27.3 ± 3.0	0.214

“mean ± SD” for normally distributed continuous data; “median (interquartile range)” for categorical data or non-normally distributed continuous data

*SD* standard deviation, *BMI *body mass index, *FES-I *fall efficacy scale-international, *PASE-C *physical activity scale for the elderly-Chinese version, *Mini-BEST *mini-balance evaluation system test, *HK-MOCA *Montreal cognitive assessment Hong Kong version; bold *: Significant difference (*p* < 0.05)

### Between-group differences in reactive balance responses

**Fig. 5:**
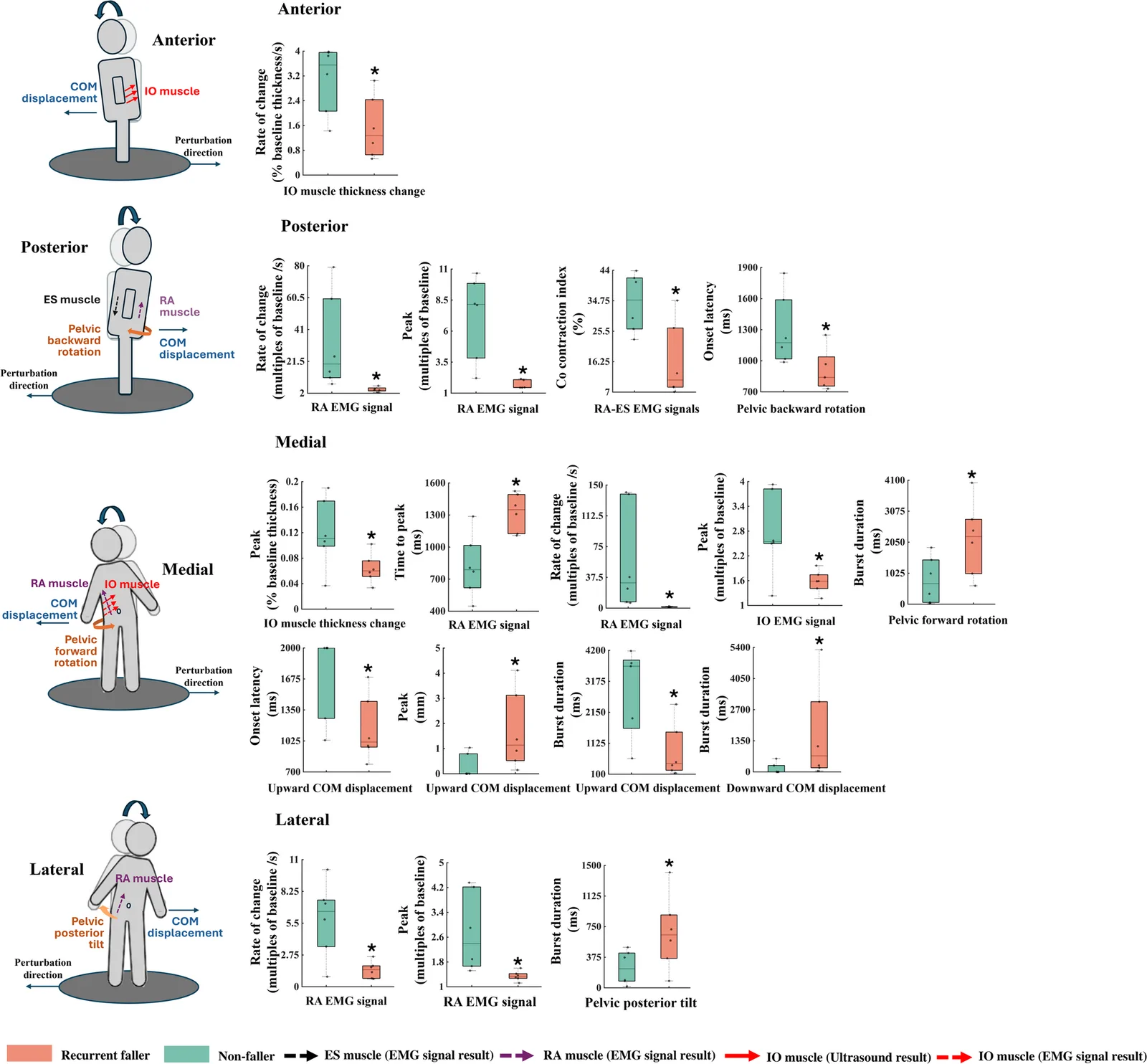
Summary of significant differences in reactive balance responses between the recurrent fallers and non-fallers. Note: RA: Rectus Abdominis; IO: Internal Oblique; ES: Erector Spinae; EMG: Electromyography; COM: Center of mass; Center line: median; Box limits: Q1 and Q3; Whiskers: min-max; bold *: Significant difference (*p* < 0.05)

All significant differences in reactive balance responses between the recurrent fallers and the non-fallers are summarized in Fig. 5; Table 2. The signal waveforms, i.e., signal changes with time, are presented in Supplementary Material Figures. The detailed between-group comparison results (including group mean and standard deviation values, percentage difference, mean difference, effect size, and *p* value) are also presented in Additional File 1.

**Table 2:** Onset differences between EMG signals and muscle thickness increases in recurrent fallers and non-fallers

Direction	Muscle	Onset difference between EMG signal and muscle thickness increase (ms)	
		Recurrent fallers (*n* = 6)	Non-fallers (*n* = 6)
Anterior	RA	27 ± 319	185 ± 373
	EO	395 ± 326	443 ± 333
	IO	339 ± 385	352 ± 393
Posterior	RA	171 ± 373	274 ± 278
	EO	465 ± 473	519 ± 349
	IO	450 ± 499	529 ± 465
Medial	RA	-194 ± 706	128 ± 391
	EO	222 ± 445	114 ± 384
	IO	296 ± 532	-45 ± 376
Lateral	RA	-504 ± 533	-101 ± 561
	EO	218 ± 357	-68 ± 315
	IO	3 ± 540	66 ± 670

Activation-contraction delay is calculated as the onset latency of muscle thickness increase subtracted by that of EMG signal. The positive value indicates that the onset latency of muscle thickness increase is larger than that of EMG signal. The negative value indicates that the onset latency of muscle thickness increase is smaller than that of EMG signal. There were no significant differences between recurrent fallers and non-fallers, nor among the muscles

*SD* standard deviation, *RA *Rectus Abdominis, *EO *External Oblique, *IO *Internal Oblique

Regarding *COM displacements*, compared to non-fallers, the recurrent fallers demonstrated significantly: (1) 33% earlier (-566 ms; *r* = 0.61; *p* = 0.034), 333% larger (1 mm; *r* = 0.60; *p* = 0.037), and 78% shorter duration (-3103 ms; d = 1.37; *p* = 0.038) of upward COM displacement, following the medial perturbations; and (2) 1069% longer duration of downward COM displacement (1518 ms; *r* = 0.61; *p* = 0.034), following the medial perturbations (Fig. 5; Supplementary Material Fig. S1). No additional significant between-group differences were observed in the remaining COM displacement parameters or for the rest three perturbation directions (Addiitonal File 1).

Regarding *dominant-side pelvic motions*, some significant differences also existed between recurrent fallers and non-fallers (Fig. 5; Supplementary Material Fig. S3). Compared to non-fallers, recurrent fallers demonstrated significantly: (1) 37% earlier pelvic backward rotation (-483 ms; d = 1.50; *p* = 0.028), following the posterior perturbations; (2) 171% longer duration for pelvic forward rotation (1357 ms; d = 1.31; *p* = 0.046), following the medial perturbations; and (3) 171% longer duration of pelvic posterior tilt (426 ms; d = 1.50; *p* = 0.043), following the lateral perturbations. No other between-group significant differences were observed in the remaining dominant-side’s pelvic motion parameters or for the other two perturbation directions (Addiitonal File 1).

Regarding *EMG signals of dominant-side trunk muscles*, recurrent fallers exhibited generally slower and reduced activation in abdominal muscles following unexpected perturbations, as compared to non-fallers (Fig. 5; Supplementary Material Fig. S5). Specifically, compared to non-fallers, recurrent fallers exhibited the significantly: (1) 82% smaller rate of EMG rise (-26.9 multiples of baseline/s; *r* = 0.69; *p* = 0.016) and 72% smaller rate of peak EMG amplitude (-5.1 multiples of baseline; *r* = 0.79; *p* = 0.006) in the RA muscle, and 53% smaller CCI (-18%; d = 1.71; *p* = 0.014) in the pair of RA and ES muscles, following posterior perturbations; (2) 97% smaller rate of EMG rise (-57.4 multiples of baseline/s; *r* = 0.83; *p* = 0.004) and 61% later time to peak EMG amplitude (501 ms; d = 2.04; *p* = 0.005) in the RA muscle, and 43% smaller peak EMG amplitude (-1.2 multiples of baseline; d = 1.62; *p* = 0.032) in the IO muscle, following medial perturbations; and (3) 74% smaller rate of EMG rise (-4.3 multiples of baseline/s; d = 1.85; *p* = 0.021) and 50% smaller peak EMG amplitude (-1.4 multiples of baseline; d = 1.55; *p* = 0.042) in the RA muscle, following lateral perturbations. No significant between-group differences were observed in the remaining EMG parameters or for the anterior perturbations (Addiitonal File 1).

Regarding *thickness changes of dominant-side trunk muscles*, recurrent fallers exhibited slower and smaller contraction in IO muscle as compared to non-fallers (Fig. 5; Supplementary Material Fig. S7). Specifically, compared to non-fallers, recurrent fallers demonstrated significantly: (1) 51% smaller rate of rise (-1.6% baseline thickness/s; d = 1.47; *p* = 0.029) of the IO muscle thickness, following anterior perturbations; and (2) 50% smaller peak (-6% baseline thickness; d = 1.32; *p* = 0.045) of the IO muscle thickness, following medial perturbations. No additional significant between-group differences were observed in the remaining thickness change parameters or for the other two perturbation directions (Addiitonal File 1).

Regarding *activation-contraction delays of dominant-side trunk muscles*, there were no significant differences in any abdominal muscles or any directions between older recurrent fallers and older non-fallers (Table 2).

### Within-group comparisons of reactive balance responses

Regarding the comparisons of *COM displacement parameters* between the two postural sway directions along an axis, a consistent pattern existed in both groups (Supplementary Material Fig. S2). Generally, for both recurrent fallers and non-fallers, the primary direction of compensatory sway (e.g., forward sway following a posterior perturbation) exhibited significantly earlier onsets and larger peak displacements, as compared to the opposing directions. Regarding group-specific observations, recurrent fallers exhibited significant differences in vertical control (upward vs. downward sway) and cross-planar coupling (e.g., forward vs. backward sway following lateral perturbations). Similarly, distinct directional differences were observed in non-fallers, especially for vertical control (upward vs. downward sway).

Regarding the comparisons of *dominant-side pelvic motion parameters* between the two motions around an axis, there was a similar pattern shared by both groups (Supplementary Material Fig. S4). Consistently, pelvic motions due to inertia (e.g., anterior tilt triggered by a backward platform shift) were significantly faster and larger than motions in the opposing direction. Regarding group-specific observations, recurrent fallers displayed significant differences in pelvic rotation (forward vs. backward rotation) and obliquity (hike vs. drop) across multiple perturbation directions. In contrast, non-fallers exhibited significant differences in pelvic tilt (anterior vs. posterior tilt) and obliquity (hike vs. drop) following mediolateral perturbations.

Regarding the comparisons of *EMG signal parameters* among the investigated dominant-side trunk muscles or muscle pairs, distinctive activation patterns were observed for the two groups (Supplementary Material Fig. S6). In recurrent fallers, significant activation differences among the trunk muscles were observed for all perturbation directions. Specifically, the RA muscle activation demonstrated later onsets, smaller rates, smaller peak amplitudes, and shorter durations, as compared to the EO/IO and ES muscles. In non-fallers, significant activation differences among the trunk muscles were identified following the lateral perturbations.

Regarding the comparisons of *thickness change parameters* among the investigated dominant-side abdominal muscles, distinct patterns were observed in the two groups (Supplementary Material Fig. S8). In recurrent fallers, significant variation was identified exclusively following medial perturbations, where the RA muscle exhibited a smaller peak thickness change as compared to the EO muscle. In non-fallers, significant differences were observed for both the medial and lateral perturbations. Specifically, the RA muscle demonstrated a smaller peak thickness change as compared to the IO muscle following medial perturbations, and later thickness change as compared to the EO muscle following lateral perturbations.

Regarding the comparisons of *activation-contraction delays* among the investigated dominant-side abdominal muscles, no significant differences were observed in any perturbation direction (Table 2).

## Discussion

By using the wearable dynamic ultrasound imaging technology (or sonomyography, SMG), this study investigated the fall-history related morphological and neuromuscular changes of trunk muscles for maintaining reactive balance in older people. Partially in line with our hypotheses, compared to non-fallers, recurrent fallers demonstrated slower and smaller activities in the IO and RA muscles following the various perturbations; however, no significant between-group differences were found for trunk muscle activation-contraction delays following perturbations. These findings may offer some initial but preliminary insights into how trunk muscles contribute to maintaining reactive balance in older recurrent fallers, especially from the perspective of muscle contractile properties. This study also supports the feasibility of utilizing wearable ultrasound imaging to evaluate muscle contraction in dynamic balance-control tasks, which might inspire and facilitate the future development of some fall-risk assessment and fall-prevention training strategies.

### Trunk muscle contraction: effects of fall histories or muscles

This study observed that among the three investigated trunk muscles, only the IO muscle contraction pattern was significantly different between older recurrent fallers and older non-fallers, in response to the unexpected anterior and medial perturbations. As far as the authors know, no prior studies have reported dynamic morphological changes of trunk muscles for maintaining reactive balance. The results of this study underscored the restrained ability and slowed reaction of IO muscle contraction, to counteract the loss of balance in recurrent fallers. The fibers of the IO muscle originate from the lateral and inferior aspects, running diagonally toward the front, medial, and superior aspects [60]. Based on this architecture, it is plausible that to recover from the backward trunk tilting, due to inertia following anterior perturbations, older participants might rely on quickly contracting their ventral trunk muscle (e.g., IO muscle) for restoring/recovering balance. When reacting to lateral balance losses that were induced by the medial perturbations, the dominant-side IO muscle could theoretically serve as the agonist muscle, pulling the pelvis medially and avoiding excessive lateral COM displacement. Given the fiber orientation and function of IO muscle, the observed slowed and reduced IO muscle thickening in older recurrent fallers might possibly represent a potential mechanistic link to their impaired ability to maintain reactive balance.

It was also interesting to observe that older recurrent fallers had a different abdominal muscle contraction sequence, when reacting to unexpected lateral perturbations, as compared to non-fallers. In contrast to older non-fallers who contracted the EO muscles earlier than the RA muscles following lateral perturbations, the older recurrent fallers contracted the two muscles simultaneously. The older non-fallers’ strategy in response to the medial balance losses seemed to be more effective than older recurrent fallers. The fibers of the EO muscle originate from the medial and inferior aspects, running diagonally toward the lateral and superior aspects [60]. Theoretically, the EO muscle, which was utilized primarily in non-fallers, might utilize the fibers orientation to pull the pelvis laterally and help them recover from the losses of the medial balance following lateral perturbations. However, recurrent fallers might not able to solely rely on the EO muscle contraction and needed additional assistance from the RA muscle to restore postural balance, potentially implying for their declined/reduced muscle quantity or quality [61, 62].

### Trunk muscle activation: effects of fall histories or muscles

Among the EMG signals of the investigated trunk muscles, the dominant-side’s IO muscle and the RA muscle were significantly different between older recurrent fallers and older non-fallers following the perturbations. On the one hand, older recurrent fallers exhibited the limited electrophysiological activation ability, echoing the above discussed morphological finding that recurrent fallers’ IO muscle contraction was limited following the medial perturbations. Given the fiber orientation of IO muscle, it was postulated that this muscle could help pull the pelvis upward to avoid excessive pelvic drop and excessive downward COM displacement [60]. Recurrent fallers’ limited activation and contraction of IO muscle was possibly relevant to their longer duration of downward COM displacement following medial perturbations, as compared to non-fallers. Previous studies also observed a similar strategy of lowering COM height (or suspensory strategy) in response to the moving-platform perturbations [20, 63, 64], and reported that this strategy could be ineffective as it led to more time for balance recovery [20]. On top of the prior research that only investigated the roles of leg muscles [20], this study suggested a potential further contribution of trunk muscles to the balance recovery strategy of lowering COM.

On the other hand, this study also observed that the RA muscle in recurrent fallers exhibited limited electrophysiological activation ability and slowed reaction, in response to the posterior and mediolateral perturbations. Following lateral balance losses induced by the medial perturbations, except for dominant-side IO muscle (serve as prime mover, with smaller activation in recurrent fallers), the dominant-side RA muscle (with slower activation in recurrent fallers) might serve as stabilizer to help maintain the core stability (avoiding excessive dominant-side trunk forward rotation) during the control of trunk rotation [65]. Following medial balance losses induced by the lateral perturbations, the dominant-side RA muscle activation (with smaller and slower activation in recurrent fallers) might contribute to counteracting the excessive trunk tilt to the non-dominant side. Moreover, the activation of RA muscle played a crucial role in maintaining the stability of pelvic anterior and posterior tilting [66–68]. Following the forward balance losses induced by posterior perturbations, it was postulated that this mechanism could serve to prevent excessive pelvic anterior tilt (with smaller activation of RA muscle and smaller co-activation level of RA-ES muscle pair in recurrent fallers).

However, inconsistent with the findings of a previous study [15], the current study identified a fall-history difference in RA muscle for maintaining reactive balance. Prior research reported that the EMG integrals of RA muscle in fallers did not significantly differ from those in non-fallers following lateral shoulder-impact perturbations [15]. Such discrepancy could be primarily due to the different types of perturbations. The shoulder-level perturbations and foot-level perturbations tended to trigger different muscle activation patterns and balance maintenance strategies [20, 49, 56, 69, 70]. Additionally, the use of different EMG parameters may have also affected the results. While previous studies mainly used the EMG integral over a period to represent the overall muscle activity, the more comprehensive and in-depth analysis of temporal and amplitude parameters (e.g., onset latency, rate of rise, time to peak, peak value, burst duration, and CCI) in the current study may have provided a more comprehensive picture of fall-history related differences.

Additionally, the current study observed some activation differences among the trunk muscles following unexpected perturbations, which might be relevant to the difference in pelvic motions around an anatomical axis or postural sways along an axis. Following the lateral trunk tilt that was induced by medial perturbations and given the fiber orientations of RA and IO muscles, it was postulated that the recurrent fallers’ slower and smaller activation in these muscles (as compared to EO and ES muscles) was possibly relevant to their inability to prevent the earlier pelvic anterior tilt (as compared to posterior tilt), earlier pelvic drop (as compared to hike), and earlier downward COM displacement. This implied that older recurrent fallers’ activation pattern of trunk muscles could be ineffective to resist the unexpected lateral balance losses.

### Trunk muscle activities and potential neural control

The observed muscle weakness (the speed, amplitude or the sequence of muscle activation/contraction) in recurrent fallers might not be solely attributed to muscle atrophy, some other neurological and non-muscular factors were also crucial in the development of muscle weakness [71–73]. Previous research reported that although muscle mass might maintain over time, older people’s muscle strength still significantly declined with aging [74]. Furthermore, older adults with leg extensor weakness exhibited significant deficits in the ability of their nervous system to fully activate the leg extensor muscles, but such neural impairments were not observed in the stronger older adults [75, 76].

According to our preliminary result, the slower rate of rise and smaller peak amplitude of RA or IO muscle activation/contraction that were observed in recurrent fallers following various perturbations, might reflect a decline in motor unit recruitment efficiency [77]. This interpretation was based on previous research suggesting that the quantity of motor unit recruitment and motor neuron discharge rates were potential determinants of the rate and peak amplitude of muscle activation/contraction [78, 79]. Furthermore, the smaller CCI of the RA and ES muscle following posterior perturbations, along with the simultaneous contraction of the EO and RA muscle following lateral perturbations in the recurrent fallers, might imply impaired inter-muscular coordination [80–82]. Previous study indicated that such decline in muscle coordination was likely the result of multifactorial interactions [82]. In addition to age-related loss of muscle mass [83], the reduced cognitive regulation capabilities due to neurodegeneration and the loss of neuromuscular network connectivity were also considered potential driving factors [82].

However, it must be emphasized that the interpretations regarding these underlying neural mechanisms should be made with caution, as the present study did not involve any direct neural measurements or related data analyses [77, 81, 82]. Future research should incorporate neuroimaging techniques, such as functional near-infrared spectroscopy (fNIRS) or electroencephalography (EEG), to provide more direct evidence of possible central neural control deficits in recurrent fallers.

### Trunk muscle activation and contraction: EMG vs. ultrasound imaging data

It is worth noting that the EMG data and the muscle thickness changes that could differentiate recurrent fallers from non-fallers were not the same. While only the IO muscle thickness change indicated a difference between the two groups, the between-group differences for EMG signals existed in both the RA and IO muscles. This inconsistent result may be attributed to three potential reasons. Firstly, the detected surface EMG signal of a specific muscle could be confounded by the crosstalk from adjacent or deep-layer muscles, especially in densely grouped areas such as the abdominal and lower back muscles [84]. The surface EMG signal amplitude is also vulnerable to the amount of adipose tissue. The muscle activities detected by the surface EMG and ultrasound imaging could be therefore different. Secondly, the EMG data may not necessarily be correlated with muscle thickness changes. A previous study has observed that the abdominal muscle thickness values were only correlated with EMG amplitudes when the intensity was less than 20% of the maximum voluntary isometric contraction (MVIC) level [85]. Thirdly, the expansion and contraction of abdominal wall, due to breathing activity, may also interfere with the trunk muscle’s active contraction in response to the unexpected perturbations. Unlike the leg muscle activities in which the dynamic ultrasound imaging commonly had more significant unique features than that from EMG signals [26–28], this study observed a greater number of significant findings from EMG data than that from ultrasound imaging data in trunk muscles. While this study has tried to filter the noise from breathing activity, it shall be noted that it has remained unclear and been difficult to determine whether the observed muscle thickness changes were free from the influence of breathing activity. Further efforts are still needed to eliminate or differentiate the effect of natural breathing on trunk muscle’s active contraction in dynamic conditions. Regardless of the exact underlying mechanics, these observed unique phenomena generally indicate that EMG and ultrasound imaging depict different perspectives of muscle function, possibly explaining why the fall-history related neuromuscular alterations were inconsistent with the morphological alterations during reactive balance control.

Contrary to our hypothesis, this study observed that the activation-contraction delays of abdominal muscles following unexpected balance perturbations did not distinguish the older recurrent fallers from older non-fallers, nor did they differ among muscles. These null findings might be due to the current ultrasound imaging equipment not being optimized to detect small physiological delays, which underpowered the results. Firstly, the frame rate of the ultrasound imaging (i.e., 20 Hz) provided a limited temporal resolution to identify the onset of muscle thickening. This might cause a deviation of up to 50 ms, making it difficult to distinguish the short delays (20–40 ms) that were reported in previous ultrafast ultrasound studies [22, 24, 25]. Secondly, unlike prior research that tracked echo intensity [21–25], the onset of muscle “thickening” was identified by using B-mode imaging. This approach may miss earlier regional activities that precede the whole-muscle deformation, further obscuring the true onset. Thirdly, the task or the type of muscle contraction should be considered [21]. Prior research reported that the EMG onset was significantly earlier than the onset of contraction as measured by ultra-fast ultrasound imaging (e.g., SMMG) during the isometric contraction of ankle dorsiflexor [22] or knee extensor [24, 25]. In contrast, the current study did not identify the significant activation-contraction delay, suggesting that the muscle activities for maintaining reactive balance do not equate the voluntary activities of a single muscle. For example, following forward balance losses induced by the posterior perturbations, the abdominal muscles were not the agonist muscles to resist perturbations, and they therefore might not exhibit a consistent activation-contraction pattern. Consequently, the combination of low temporal resolution, onset definition differences and different task/type of muscle contraction suggested that the current protocol was not sensitive enough to confirm the hypothesized activation-contraction delays.

### Implications for future clinical practice and research perspectives

This study supported the feasibility of using wearable B-mode ultrasound imaging of skeletal muscles to evaluate reactive balance performance and differentiate fall risks in older adults. Given the observed fall-history related alterations in abdominal muscle activation and contraction patterns, this study preliminarily identified the importance of specific IO, RA, and EO muscles for fall-prone older people to maintain reactive balance. Based on these preliminary findings, future research is warranted to quantify the diagnostic accuracies (e.g., sensitivity, specificity, area under the curve) of these muscle morphological parameters. Such validation is a necessary step before determining if this technology is suitable for clinical fall-risk assessment. Additionally, with the feasible and validated application of wearable dynamic ultrasound imaging during perturbations, this technology and the identified fall-related morphological parameters might be used to evaluate the effects of training that target reactive balance (e.g., perturbation-based balance training) in future attempts [86].

The findings of this study could also inspire future clinical practice in terms of developing some targeted IO, RA, or EO training programs for older recurrent fallers, which might help enhance their reactive muscle activation and contraction following balance perturbations or even fall risks. For example, the IO muscle could be focused to improve fall-prone older people’s strategies for resisting backward and lateral balance losses, the RA muscle could be trained to improve their strategies for resisting forward and mediolateral balance losses, and the high-speed resistance training of EO muscle could be done to improve their contraction sequence of abdominal muscles following medial balance losses. However, randomized controlled trials are needed to verify whether these strengthening strategies can effectively translate to improved reactive balance recovery. Furthermore, although real-time ultrasound visual feedback muscle strength training has shown promise in stroke patients [27], it remains to be investigated whether incorporating this biofeedback into abdominal muscle training can lead to superior reductions in fall risk compared to traditional methods.

### Study limitations

This study has several limitations requiring future efforts and research for further optimization. Firstly, the use of convenience sampling, strict exclusion criteria, small size and gender-unbalanced sample limits the generalization of findings. The cross-sectional design precludes causal inferences between muscular alterations and fall history. It is therefore noteworthy that the mechanistic explanations linking specific fiber orientations of abdominal muscles to fall-related kinematic differences are just based on the anatomy and speculative. Future research should aim to validate the current findings in larger, more diverse cohorts using longitudinal designs to unravel the causality between muscle morphological characteristics and fall risk. Secondly, the wearable B-mode ultrasound imaging technology merits further development to improve its temporal resolution, which will provide a more accurate depiction of morphological mechanisms and activation-contraction delays. Thirdly, although post-hoc filtering was applied to manage breathing artifacts, independent respiratory monitoring (e.g., IMUs) is merited in future attempts to more accurately differentiate trunk muscle contractions caused by breathing from those caused by perturbations. Finally, this study focused only on dominant-side trunk muscles and only on abdominal ultrasound, normalized the EMG or thickness change signal based on unperturbed standing, and extracted the discrete signal parameters for analysis. This approach may overlook the bilateral asymmetries, the relevance to individual’s maximal capability, and the complex temporal patterns of muscle activities for maintaining reactive balance. Future research can extend bilateral trunk muscles assessment, use the MVIC value for normalization, and try continuous waveform analysis.

## Conclusions

This pilot study observed the older recurrent fallers’ slower and/or smaller activation and contraction of abdominal muscles following unexpected moving-platform perturbations, as compared to older non-fallers. These alterations were specific to the direction of perturbations, which might be potentially relevant to the specific fiber orientation and function of RA, IO, and EO muscles. Specifically, compared to older non-fallers, older recurrent fallers exhibited: (1) insufficient IO muscle contraction in response to sudden backward/lateral balance losses, (2) insufficient IO muscle activation following sudden lateral balance losses, and (3) insufficient RA muscle activation following sudden forward/medial/lateral balance losses. Additionally, non-fallers prioritized the activation and contraction of EO muscle before those of RA muscle to resist sudden medial balance losses, while recurrent fallers did not show this sequence of muscle activities. These reactive muscular alterations might be relevant to the recurrent fallers’ inability to resist perturbation-induced pelvic motions and non-fallers’ shorter duration of balance recovery. Altogether, this study preliminarily demonstrated the feasibility and appropriateness of using wearable dynamic ultrasound imaging of trunk muscles to characterize morphological alterations that were related to fall history in older people. Given the preliminary findings, further research with larger and adequately powered samples is required to confirm their value for fall-risk assessment.

## Supplementary Information

Supplementary Material 1.

Supplementary Material 2: Fig S1. The COM displacements of 6 recurrent fallers (mean ± SD) and 6 non-fallers (mean ± SD) following unexpected moving-platform perturbations. Note: SD: Standard Deviation; COM: Center of Mass. Fig S2. The significant within-group comparison results in COM displacement parameters. Note: F: Forward; B: Backward; M: Medial; L: Lateral; COM: Center of Mass. Fig S3. Dominant-side pelvic motions of 6 recurrent fallers (mean ± SD) and 6 non-fallers (mean ± SD) following unexpected moving-platform perturbations. Note: SD: Standard Deviation. Fig S4. The significant within-group comparison results in dominant-side pelvic motion parameters. Note: A: Anterior; P: Posterior; H: Hike; D: Drop; F: Forward; B: Backward. Fig S5. EMG signals of dominant-side trunk muscles of 6 recurrent fallers (mean ± SD) and 6 non-fallers (mean ± SD) following unexpected moving-platform perturbations. Note: SD: Standard Deviation; RA: Rectus Abdominis; EO: External Oblique; IO: Internal Oblique; ES: Erector Spinae. Fig S6. The significant within-group comparison results in EMG signal parameters of dominant-side trunk muscles. Note: RA: Rectus Abdominis; EO: External Oblique; IO: Internal Oblique; ES: Erector Spinae. Fig S7. Thickness changes of dominant-side trunk muscles of 6 recurrent fallers (mean ± SD) and 6 non-fallers (mean ± SD) following unexpected moving-platform perturbations. Note: SD: standard deviation; RA: Rectus Abdominis; EO: External Oblique; IO: Internal Oblique. Fig S8. The significant within-group comparison results in thickness change parameters of dominant-side trunk muscles. Note: RA: Rectus Abdominis; EO: External Oblique; IO: Internal Oblique.

## Data Availability

The original contributions presented in this study are included in the article. Further inquiries can be directed at the corresponding authors.

## Abbreviations

CCI: Co-contraction index
COM: Center of mass
COP: Center of pressure
EMG: Electromyography
EO: External oblique
ES: Erector spinae
FES-I: Falls efficacy scale-international
FFT: Fast fourier transform
IO: Internal oblique
Mini-BEST: Mini-balance evaluation system test
MoCA: Montreal cognitive assessment
MVIC: Maximum voluntary isometric contraction
PASE-C: Physical activity scale for the elderly-Chinese version
RA: Rectus abdominis
SD: Standard deviation
