# Correction: Different trunk muscle responses to unexpected balance perturbations between older recurrent fallers and older non-fallers: a combined wearable ultrasound imaging and electromyography (EMG) study

**DOI:** 10.1186/s12877-026-08224-w

**Published:** 2026-09-15

**Authors:** Hao-Bin Liang, Ringo Tang-Long Zhu, Yu-Yan Luo, Zhen Song, Wei-Tao He, Isabella Chee-Yin Yam, Ka-Shing Lee, Wei Ren, Ke-Jing Li, Feng-Yi Wang, Shuai Li, Huiying Cynthia Hou, Quan Wei, Yong-Ping Zheng, Xiu-Qin Ye, Christina Zong-Hao Ma

**Affiliations:** 1 Department of Biomedical Engineering, The Hong Kong Polytechnic University, Hung Hom, 999077 Hong Kong SAR China; 2 Research Institute for Smart Ageing, The Hong Kong Polytechnic University, Hung Hom, 999077 Hong Kong SAR China; 3 Department of Rehabilitation, Southwest Hospital, Third Military Medical University (Army Medical University), Chongqing, 400038 China; 4 Department of Rehabilitation Sciences, The Hong Kong Polytechnic University, Hung Hom, 999077 Hong Kong SAR China; 5 Department of Computer Science, City University of Hong Kong, Hung Hom, 999077 Hong Kong SAR China; 6 Rehabilitation Medicine Center, Institute of Rehabilitation Medicine, West China Hospital, Sichuan University, Chengdu, 610041 China; 7 Key Laboratory of Rehabilitation Medicine in Sichuan Province, West China Hospital, Sichuan University, Chengdu, 610041 China; 8 Department of Building Environment and Energy Engineering, The Hong Kong Polytechnic University, Hung Hom, Hong Kong SAR China; 9 Ultrasound Department, Shenzhen People’s Hospital, The Second Clinical Medical College of Jinan University, Shenzhen, 518020 China

**Correction: BMC Geriatr 26, 368 (2026)**

**https://doi.org/10.1186/s12877-026-07158-7**

Following publication of the original article [1], the authors identified an error in the author name of Ka-Shing Lee.

The incorrect author name is: Ka-Shing Li

The correct author name is: Ka-Shing Lee

The author group has been updated above and the original article [1] has been corrected.
