# Supplementary material for: Different trunk muscle responses to unexpected balance perturbations between older recurrent fallers and older non-fallers: a combined wearable ultrasound imaging and electromyography (EMG) study
Source: BMC Geriatr. 2026 Feb 12;26:368. doi: 10.1186/s12877-026-07158-7 (PMC12998121; doi:10.1186/s12877-026-07158-7)
Supplement: Supplementary file 2 — Supplementary Material 2: Fig S1. The COM displacements of 6 recurrent fallers (mean ± SD) and 6 non-fallers (mean ± SD) following unexpected moving-platform perturbations. Note: SD: Standard Deviation; COM: Center of Mass. Fig S2. The significant within-group comparison results in COM displacement parameters. Note: F: Forward; B: Backward; M: Medial; L: Lateral; COM: Center of Mass. Fig S3. Dominant-side pelvic motions of 6 recurrent fallers (mean ± SD) and 6 non-fallers (mean ± SD) following unexpected moving-platform perturbations. Note: SD: Standard Deviation. Fig S4. The significant within-group comparison results in dominant-side pelvic motion parameters. Note: A: Anterior; P: Posterior; H: Hike; D: Drop; F: Forward; B: Backward. Fig S5. EMG signals of dominant-side trunk muscles of 6 recurrent fallers (mean ± SD) and 6 non-fallers (mean ± SD) following unexpected moving-platform perturbations. Note: SD: Standard Deviation; RA: Rectus Abdominis; EO: External Oblique; IO: Internal Oblique; ES: Erector Spinae. Fig S6. The significant within-group comparison results in EMG signal parameters of dominant-side trunk muscles. Note: RA: Rectus Abdominis; EO: External Oblique; IO: Internal Oblique; ES: Erector Spinae. Fig S7. Thickness changes of dominant-side trunk muscles of 6 recurrent fallers (mean ± SD) and 6 non-fallers (mean ± SD) following unexpected moving-platform perturbations. Note: SD: standard deviation; RA: Rectus Abdominis; EO: External Oblique; IO: Internal Oblique. Fig S8. The significant within-group comparison results in thickness change parameters of dominant-side trunk muscles. Note: RA: Rectus Abdominis; EO: External Oblique; IO: Internal Oblique. [file 12877_2026_7158_MOESM2_ESM.docx]

**Supplementary Material**


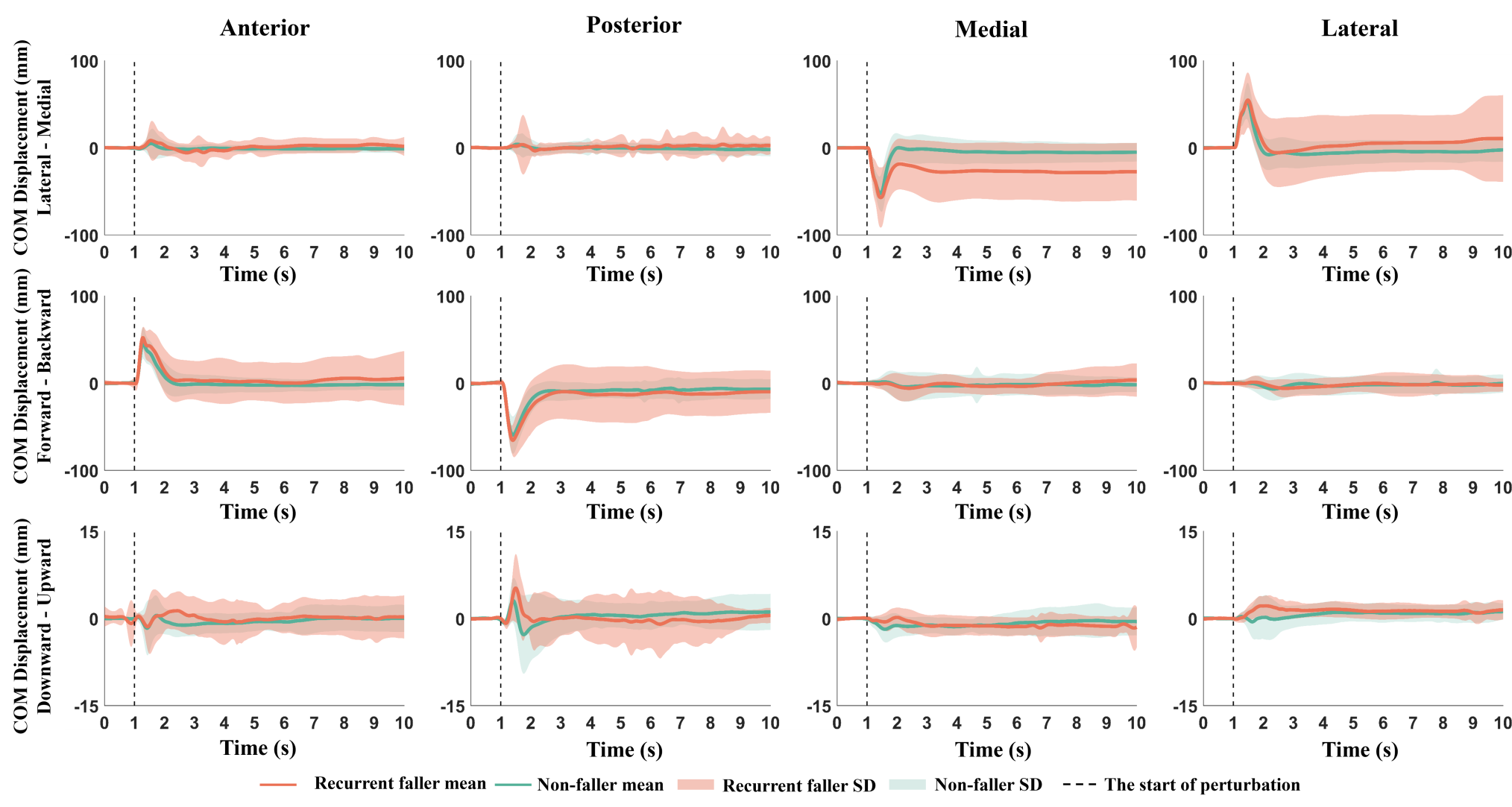


**Figure S1.** The COM displacements of 6 recurrent fallers (mean ± SD) and 6 non-fallers (mean ± SD) following unexpected moving-platform perturbations.

Note: SD: Standard Deviation; COM: Center of Mass.


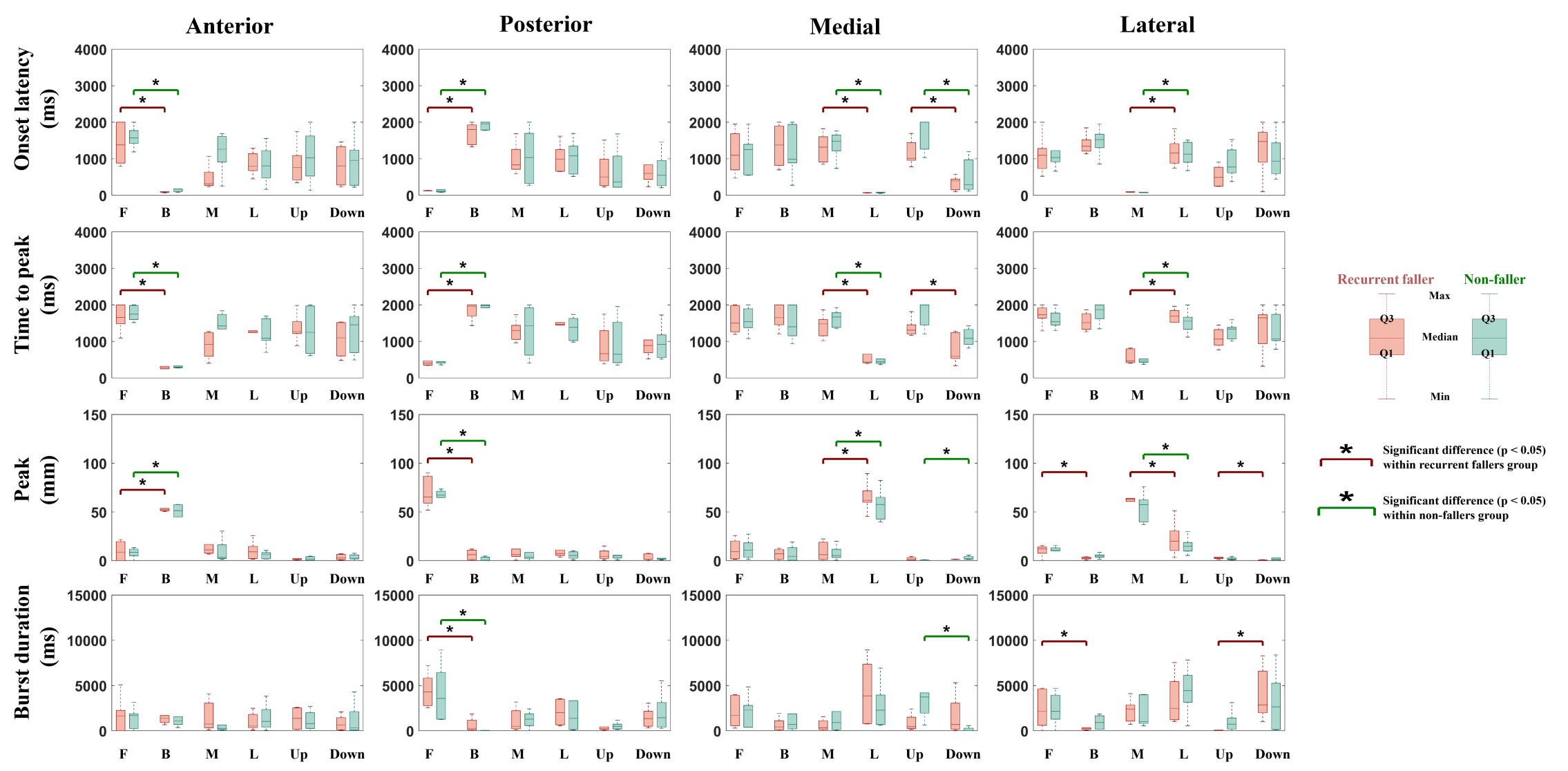


**Figure S2.** The significant within-group comparison results in COM displacement parameters.

Note: F: Forward; B: Backward; M: Medial; L: Lateral; COM: Center of Mass.


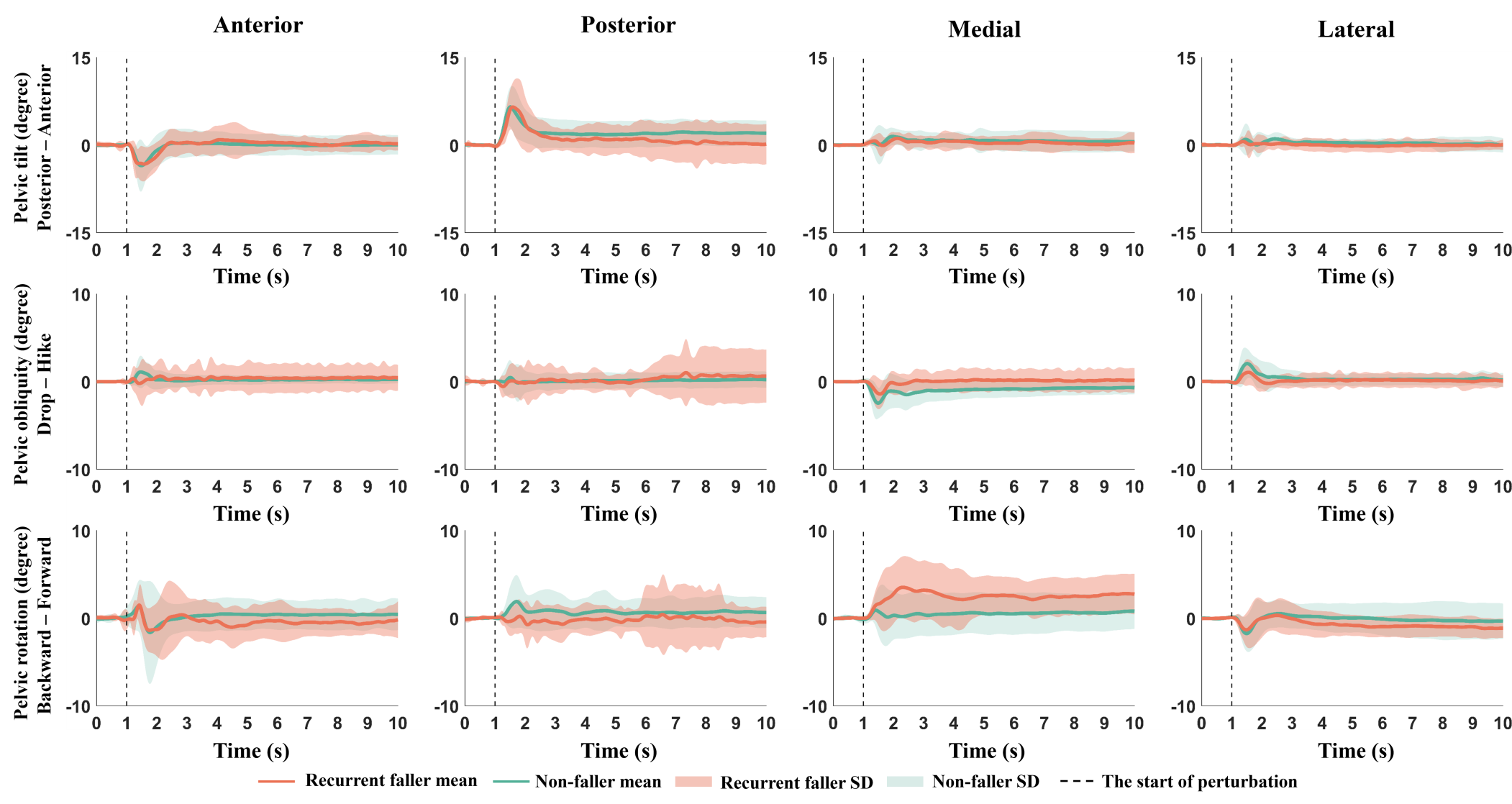


**Figure S3.** Dominant-side pelvic motions of 6 recurrent fallers (mean ± SD) and 6 non-fallers (mean ± SD) following unexpected moving-platform perturbations.

Note: SD: Standard Deviation.


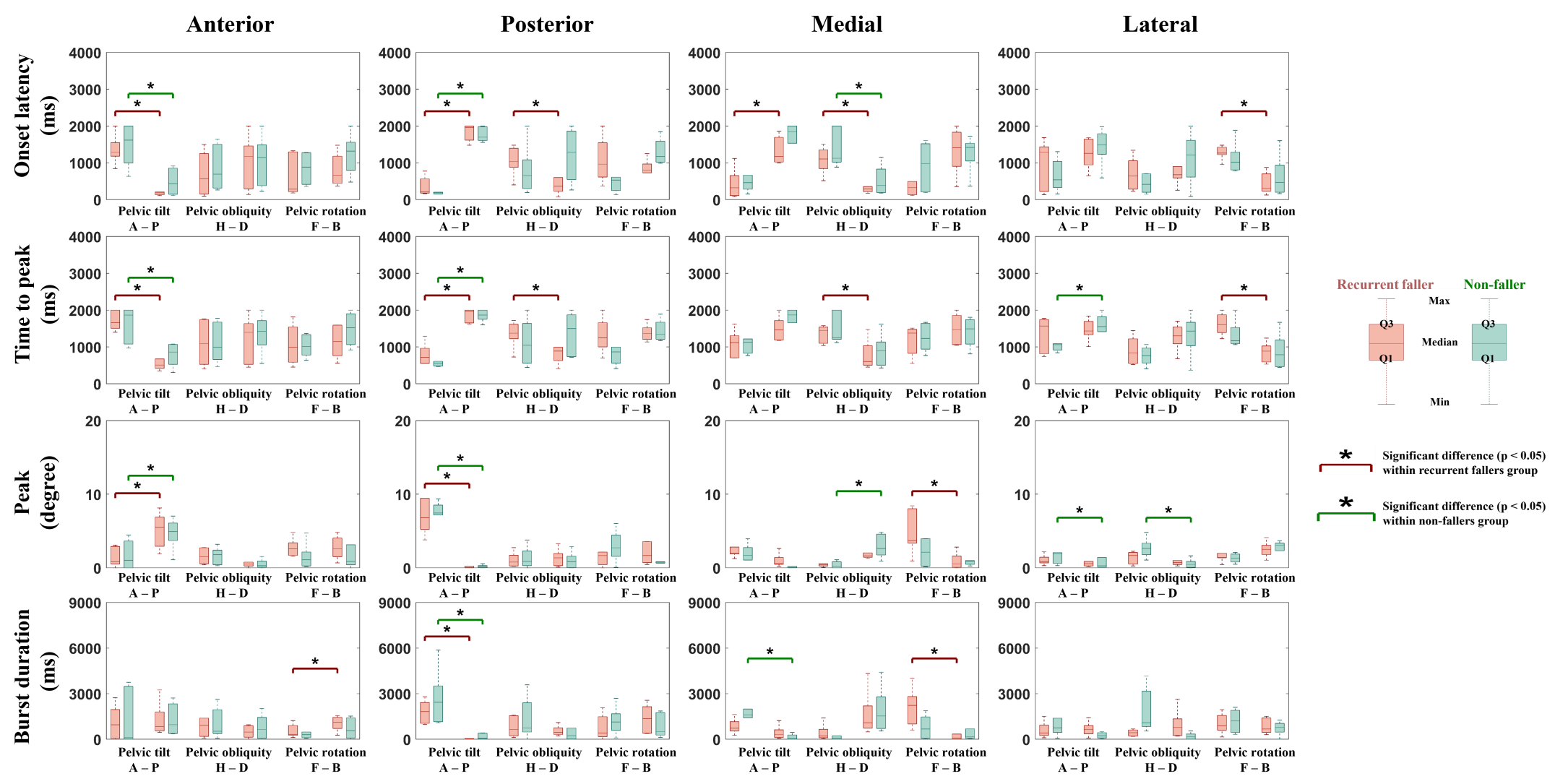


**Figure S4.** The significant within-group comparison results in dominant-side pelvic motion parameters.

Note: A: Anterior; P: Posterior; H: Hike; D: Drop; F: Forward; B: Backward.


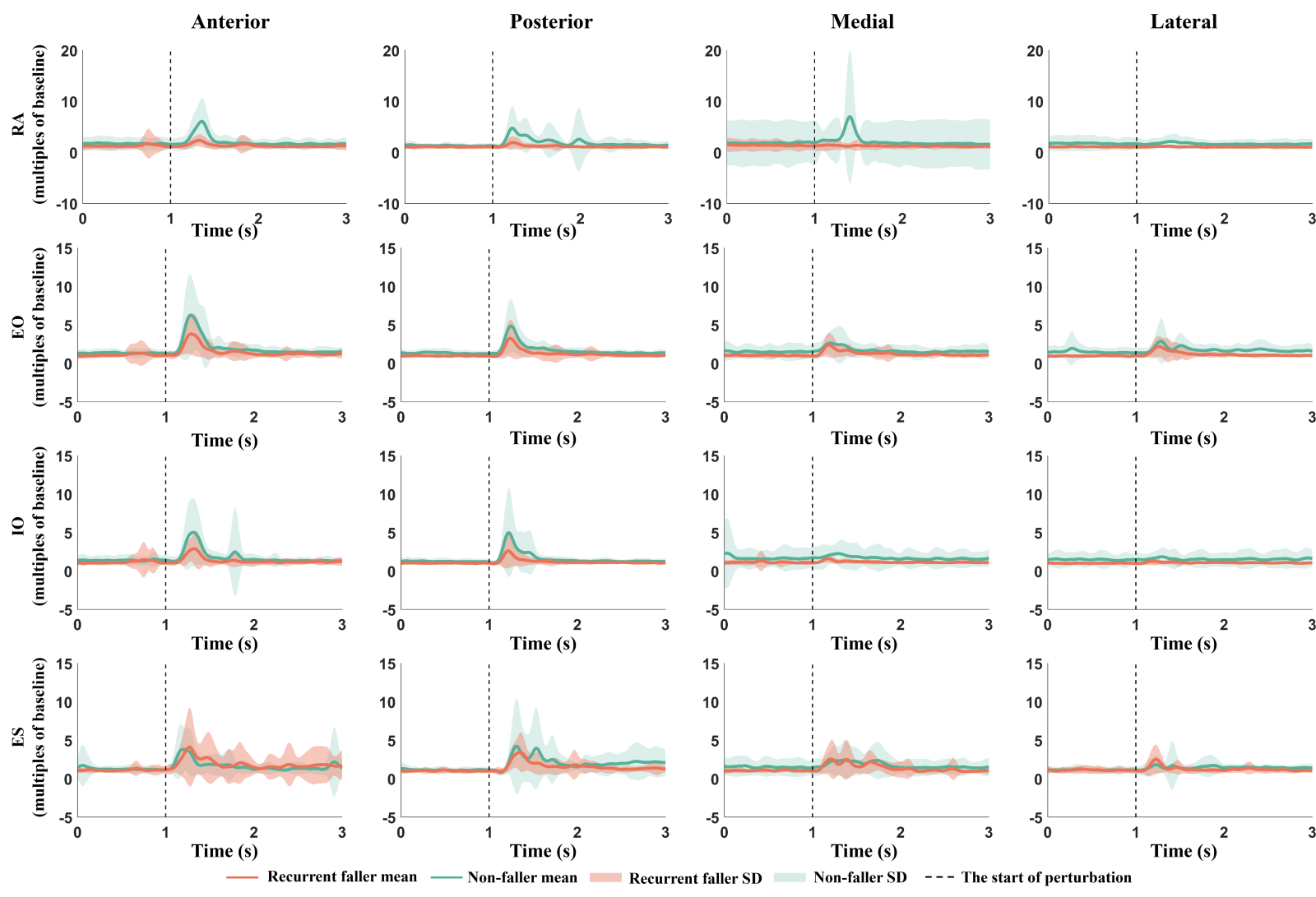


**Figure S5.** EMG signals of dominant-side trunk muscles of 6 recurrent fallers (mean ± SD) and 6 non-fallers (mean ± SD) following unexpected moving-platform perturbations.

Note: SD: Standard Deviation; RA: Rectus Abdominis; EO: External Oblique; IO: Internal Oblique; ES: Erector Spinae.


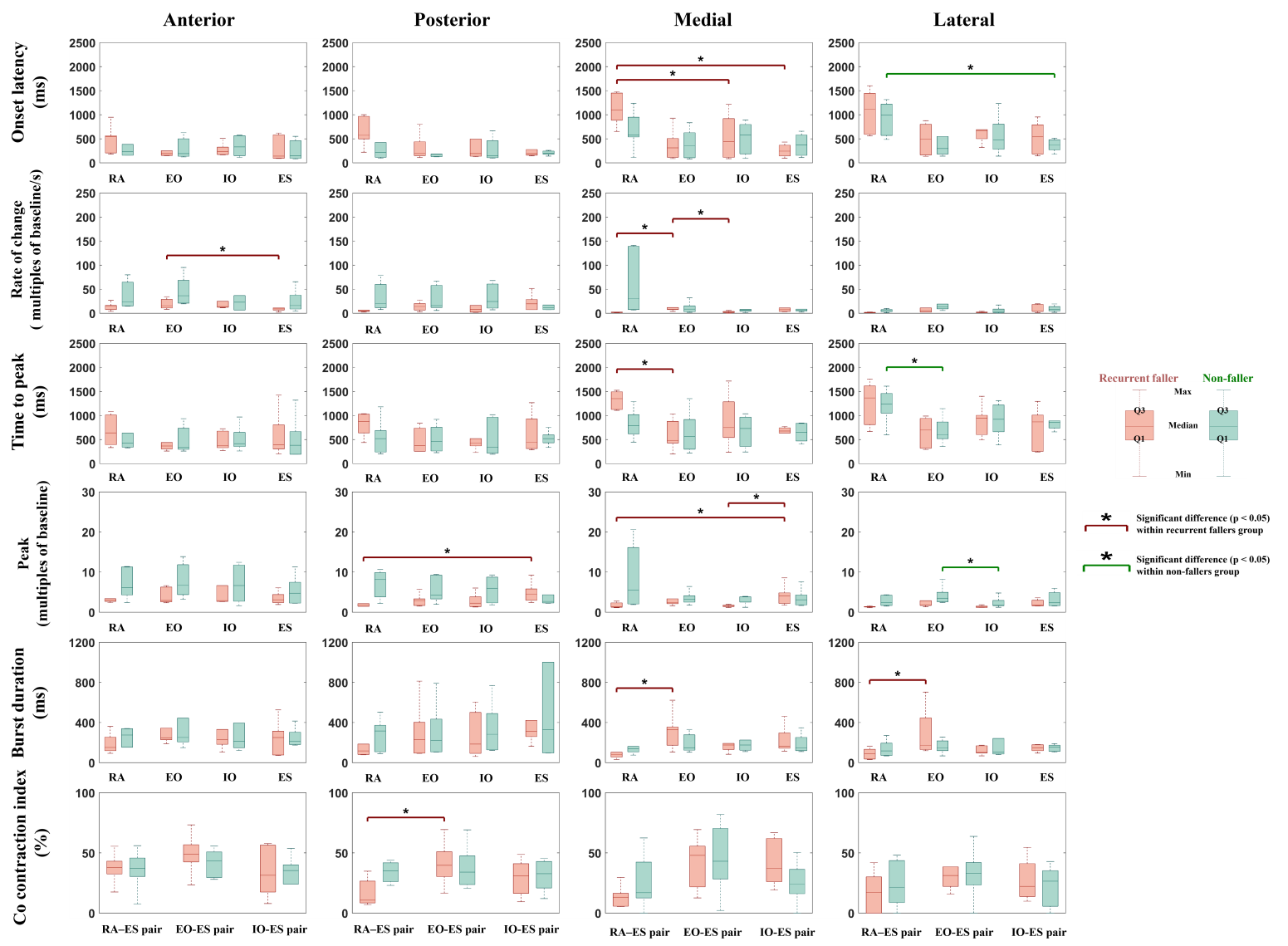


**Figure S6.** The significant within-group comparison results in EMG signal parameters of dominant-side trunk muscles.

Note: RA: Rectus Abdominis; EO: External Oblique; IO: Internal Oblique; ES: Erector Spinae.


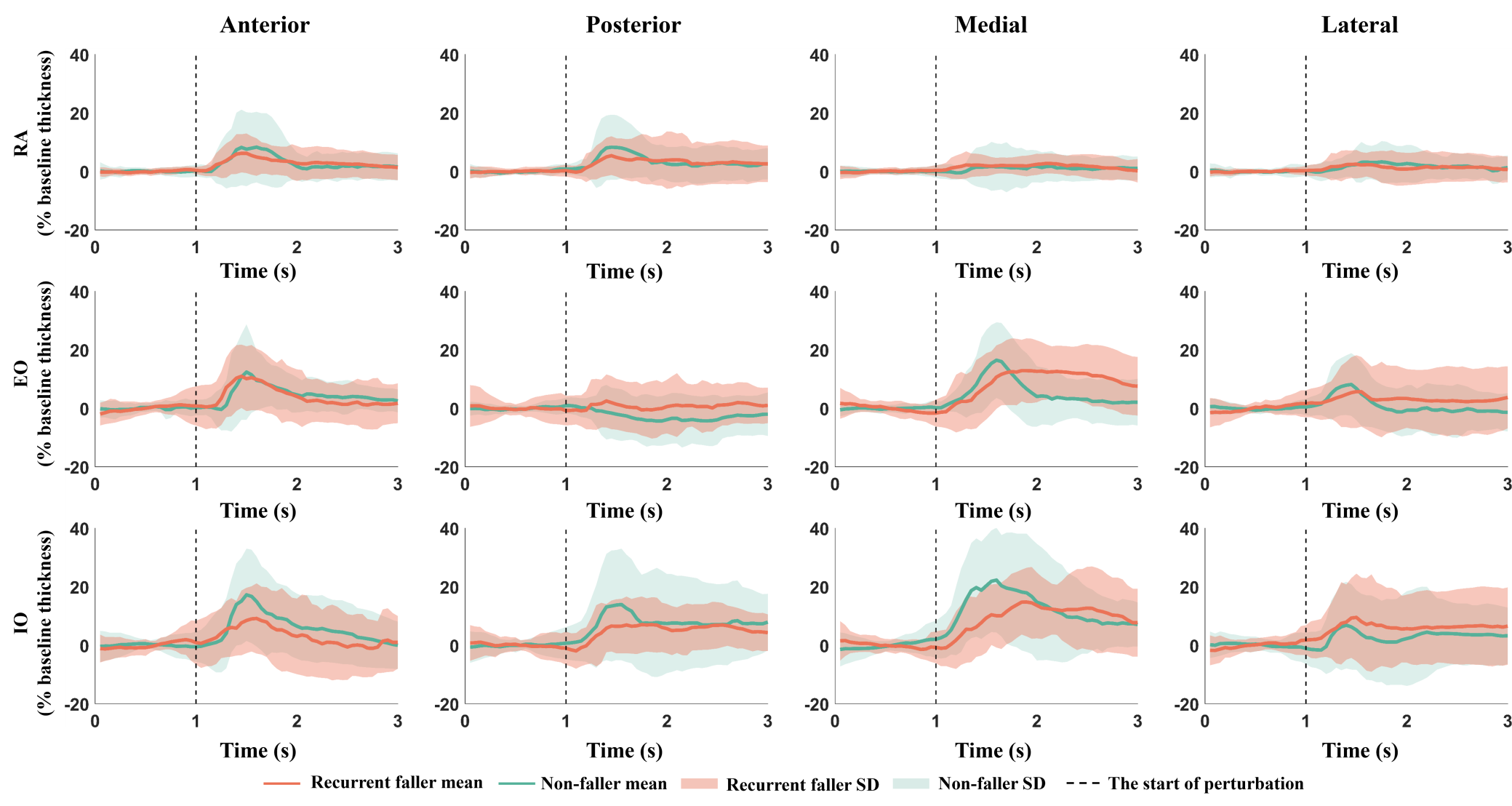


**Figure S7.** Thickness changes of dominant-side trunk muscles of 6 recurrent fallers (mean ± SD) and 6 non-fallers (mean ± SD) following unexpected moving-platform perturbations.

Note: SD: standard deviation; RA: Rectus Abdominis; EO: External Oblique; IO: Internal Oblique.


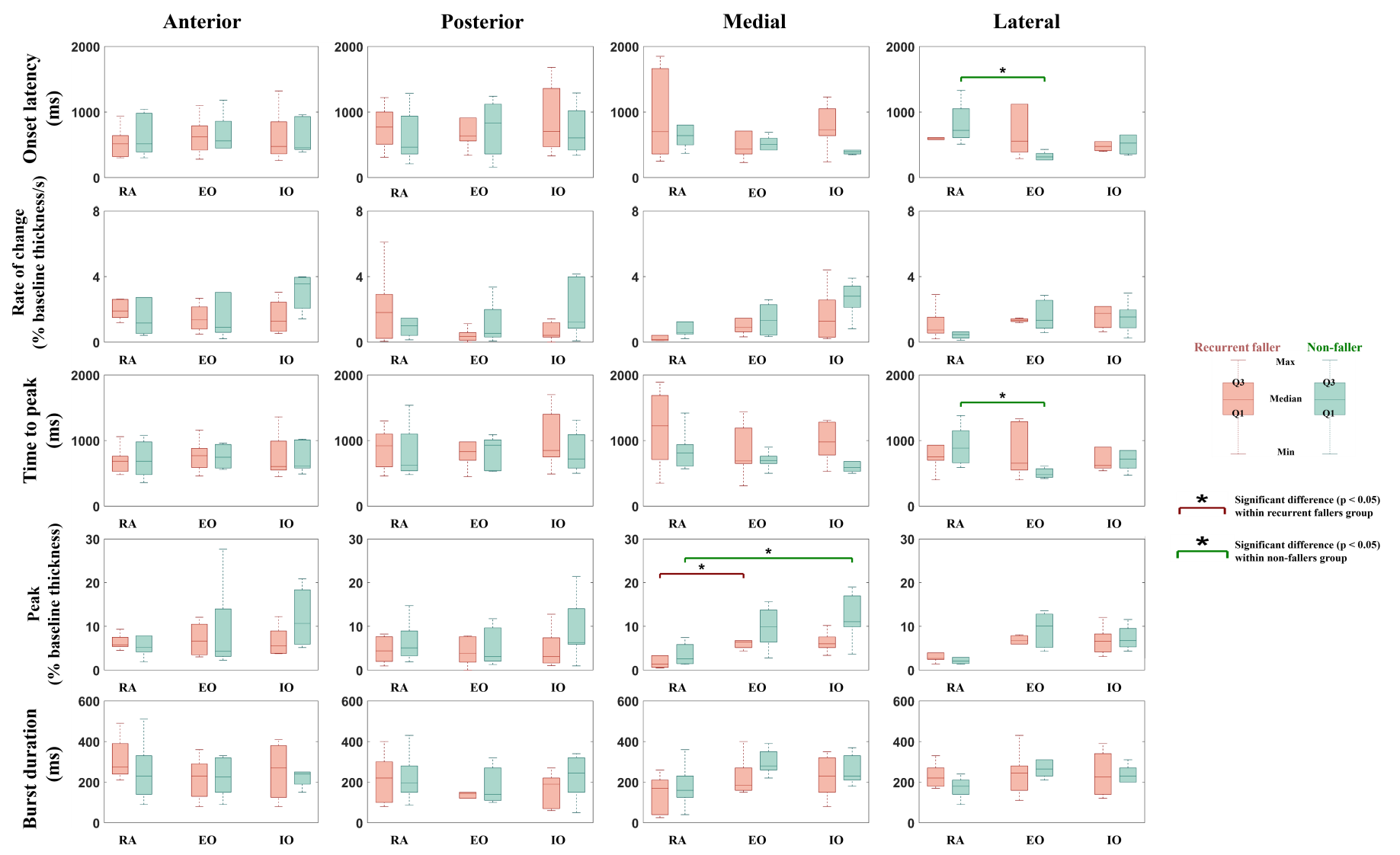


**Figure S8.** The significant within-group comparison results in thickness change parameters of dominant-side trunk muscles.

Note: RA: Rectus Abdominis; EO: External Oblique; IO: Internal Oblique.
